# Obtaining Longitudinal Built Environment Data Retrospectively across 25 years in Four US Cities

**DOI:** 10.3389/fpubh.2016.00065

**Published:** 2016-04-19

**Authors:** Jana A. Hirsch, Katie A. Meyer, Marc Peterson, Daniel A. Rodriguez, Yan Song, Ke Peng, Jun Huh, Penny Gordon-Larsen

**Affiliations:** ^1^Carolina Population Center, University of North Carolina at Chapel Hill, Chapel Hill, NC, USA; ^2^Department of Nutrition, University of North Carolina at Chapel Hill, Chapel Hill, NC, USA; ^3^Department of City and Regional Planning, University of North Carolina at Chapel Hill, Chapel Hill, NC, USA

**Keywords:** community design, field audit, transportation, infrastructure, parks, GIS, longitudinal, methods

## Abstract

**Background:**

Neighborhood transportation infrastructure and public recreational facilities are theorized to improve the activity, weight, and cardiometabolic profiles of individuals living in close proximity to these resources. However, owing to data limitations, there has not been adequate study of the influence of timing and placement of new infrastructure on health over time.

**Methods:**

This protocol details methods of the four cities study to perform retrospective field audits in order to capitalize on existing longitudinal health data from the coronary artery risk development in young adults (CARDIA) study. We developed and verified measures of recreation facilities (trails, parks) and transportation infrastructure (bus, light rail, bicycle parking, bicycle paths) in Birmingham, AL; Chicago, IL; Minneapolis, MN; and Oakland, CA (USA). We identify introductions, renovations, and closures between 1985 and 2010 to develop measures of facility and infrastructure change. Ultimately, these data were linked to CARDIA sites’ respondents’ geographic locations over the 25-year study period to examine associations with health behaviors and outcomes.

**Results:**

Data available for retrospective audits was inconsistent by city, primarily due to record-keeping differences. We found large increases in bicycle infrastructure, with the exception of Birmingham, AL, USA. Excluding the addition of a new rail line in Minneapolis, MN, USA, few changes occurred in bus service, rail, and parks.

**Conclusion:**

Our method represents innovation toward the collection of retrospective neighborhood data for use in longitudinal analyses. The data produced give insight into the way neighborhood infrastructure has changed over time and the potential relationship between these changes and health behaviors.

## Background

A large body of literature suggests that some neighborhood characteristics can influence cardiovascular health (1–4). Neighborhoods that offer access to safe and accessible recreational facilities and transportation infrastructure are theorized to improve the diet, physical activity, weight, and cardiometabolic profiles of individuals living in close proximity to these resources (5–7). Observational studies to identify the specific factors and pathways linking neighborhood environments to cardiometabolic risk factors, however, have been largely cross-sectional and have produced mixed results (8–23). The major limitations of this current body of research are selection bias and reverse causation, largely due to the faulty assumption that neighborhood resources are placed independent of all other factors and that no selective migration occurs to take advantage of these resources (24–26).

There have been dramatic changes in the US physical activity and infrastructure environments during the past few decades (27–29). Studying changes in neighborhoods over time with respect to health behaviors and outcomes could improve our understanding of how changes in environments may shape changes in behaviors, thus providing insight into the potential for community investments in infrastructure and recreational facilities. Yet, minimal longitudinal evidence exists (30–45), much of it focusing on changes due to residential relocations (33, 44, 45) rather than environmental changes around stable residents. Although policies and changes to the built environment could be evaluated as “natural” or “quasi” experiments, a recent review found only 18 studies evaluating policy or change impacts on nutrition/diet, 17 on physical activity, and 3 on body mass index, with a wide variety in the quality of the study designs (46). The largest issue is the lack of high quality, longitudinal data, and methods to study retrospective data on neighborhood infrastructure changes that could be linked to existing longitudinal cohorts of health behaviors and outcomes. This leaves fundamental gaps in our ability to study the influence of timing and placement of new infrastructure on activity behaviors, obesity, and cardiometabolic risk over time.

To this end, we created a methodology to collect, verify, and process data on dynamic infrastructure changes and quantify associations between infrastructure and health behaviors and outcomes in four US cities over a 25-year period, which we linked to high quality, longitudinal clinic data from the coronary artery risk development in young adults (CARDIA) study from 1985–1986 to 2010–2011. Specifically, we generated data to allow the development of a model to investigate how the timing and placement of changes in recreation facilities and transportation infrastructure influence: (a) individual-level physical activity, (b) patterns of weight maintenance and gain, and (c) cardiometabolic risk measures over 25 years, controlling for the purposive placement of recreation facilities and transportation infrastructure and for the selective migration of individuals to locate near such resources.

In order to accomplish this larger research goal, we developed and verified measures to capture introductions, renovations, and closures representing changes in (1) recreation facilities (e.g., trails and parks) and (2) transportation infrastructure (e.g., light rail, bike parking, and bike paths) in the NIH-funded CARDIA four cities study (R01-HL114091). We performed retrospective field audits to enhance our assessment of above mentioned facility and infrastructure data in the four original CARDIA field sites over the period from 1985–1986 to 2010–2011. In this paper, we present our protocol to perform these field audits, as well as verify and process the data collected.

## Methods/Design

**Figure 1:**
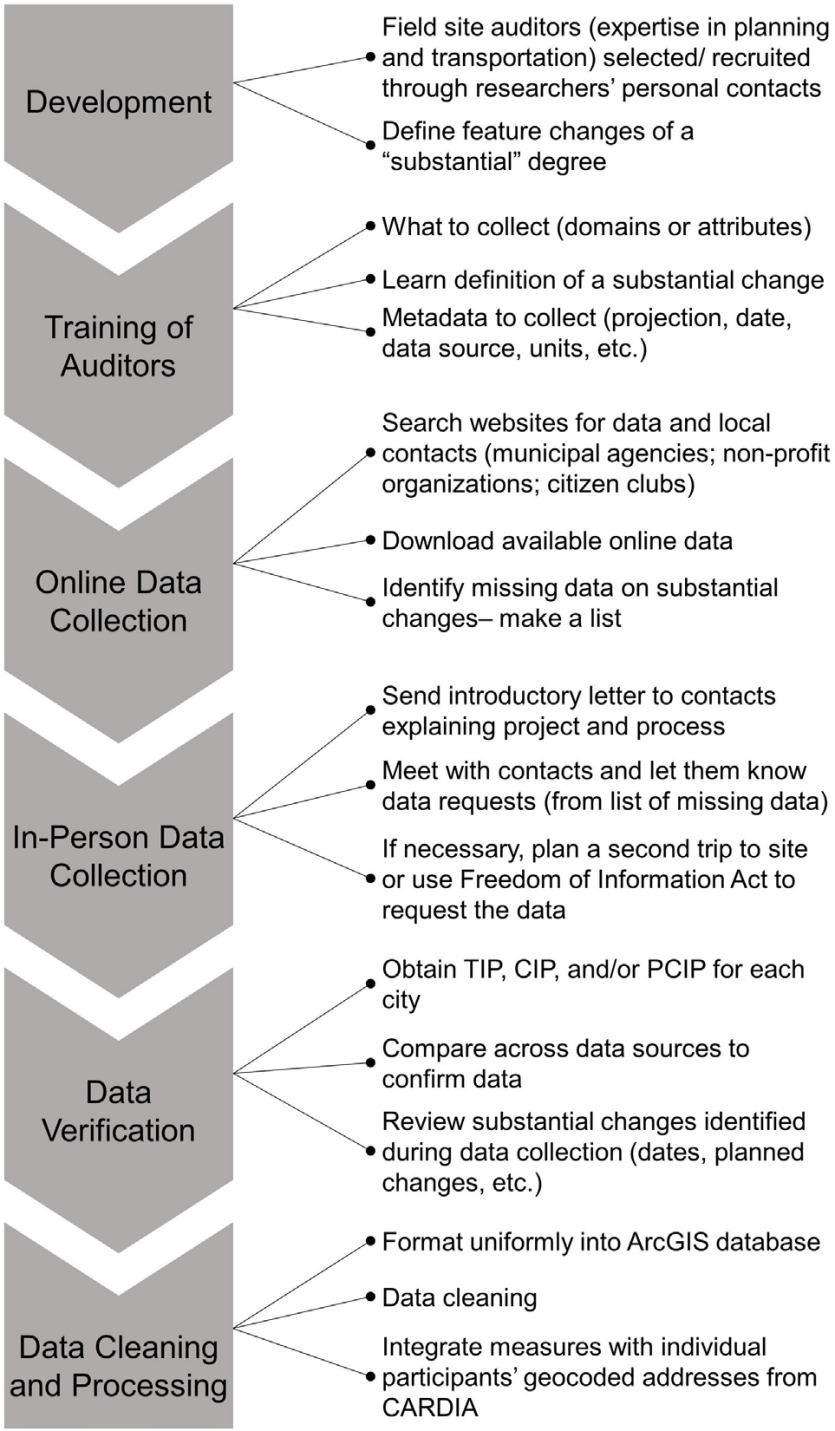
**Flow chart of the overall process used for retrospective field audits**.

A flow chart of the overall process used for retrospective field audits can be found in Figure 1.

### CARDIA Study

The four cities study is an ancillary study of the CARDIA study, a prospective cohort study initiated to investigate life-style and other factors that influence the evolution of coronary heart disease risk factors during young adulthood (47). CARDIA includes 5,115 participants, aged 18–30 years, who were recruited and examined in four urban areas: Birmingham, AL; Chicago, IL; Minneapolis, MN; and Oakland, CA (USA) (48) in 1985–1986 with 8 waves of data collection until 2010–2011. The four cities in our sample are emblematic of different types of US cities and represent three distinct geographic regions (South, Midwest, and West). Study data were collected under protocols approved by Institutional Review Boards at each study center and UNC at Chapel Hill.

### Neighborhood Definition and Coverage

In studies using geographic information system (GIS)-derived environment variables, neighborhood definitions can be generally classified into administrative boundaries [e.g., counties (49), Metropolitan Statistical Areas (50), zip codes, census tract (51), or block groups (1, 52, 53)] and buffers [e.g., Euclidian (34, 43) or street network (54, 55) distances around a point of interest like a residence or employment site]. Although these boundaries are readily and inexpensively available, they are somewhat arbitrary and may not correspond with what the population itself may consider as a neighborhood. By contrast, there are also conceptually defined neighborhoods, often used by local planners, such as the community areas created by the Social Science Research Committee at University of Chicago that has divided the City of Chicago into 77 well defined and consistent community areas (56). Analogous data are available from city planning departments for the other three cities (Birmingham: 99; Minneapolis/St. Paul 81/17; Oakland: 57 neighborhoods). In this project, we used administrative neighborhoods, Euclidean, and network buffer-based neighborhoods, as well as conceptually defined neighborhoods from the Regional Planning Commission of Greater Birmingham (Birmingham) and Zillow (Chicago, Minneapolis, and Oakland). Thus, the four cities database allows for a variety of neighborhood geographies for flexibility and tailoring relative to specific research questions. All neighborhood measures were based around the place of residence of participants.

The nominal geographic areas covered in the data collection effort were constrained to the official administrative boundaries of each of the four cities. The enclave city of Piedmont was included with the Oakland, CA, USA site, and the Norridge and Harwood Heights enclaves were included with the Chicago, IL, USA site. The particular geographic coverage of the different feature types varies to some degree, however, and some features also extend beyond each city boundary. Coverage maps of the areal extent per feature type are provided in Figures S1–S5 in Supplementary Material. Although approximately 25% of the CARDIA sample has moved outside of the four cities over the past 25 years, the four cities sample was restricted to the core four cities due to the resource intensity of the data collection process.

### Field Audit Team and Training

Field site auditors (*n* = 5; one for Oakland, Minneapolis, and Birmingham and two at UNC who collected data in Chicago and coordinated the other field auditors) were recruited through personal contacts of the researchers and selected based on experience and professional training in city/regional planning and/or transportation engineering as well as familiarity with and connections in each of the four cities. The field auditors were instructed to collect data on introductions, renovations, and closures representing changes in recreation facilities (e.g., trails and parks) and transportation infrastructure (e.g., light rail, bike parking, and bike paths) over the period from 1985–1986 to 2010–2011. In January, 2012, UNC investigators and team members hosted an in-person orientation and training for all of the four cities field auditors. The protocol was developed by the four cities study investigators and reflected state-of-the-art in built environment knowledge and methods at the time (literature, experience of investigators). Training included a background to study and study objectives, descriptions of data features sought, instruction on data sources, and study guidelines on data standards and organization, application of GIS software for data management, as well as contingencies for possible challenges in the field. During active data collection, there was a weekly conference call with all field auditors and four cities’ investigators to discuss progress, address challenges, and share successful approaches for data collection, with ongoing daily support for auditors to ensure that data collection protocols were implemented with fidelity and to aid in data collection efforts. The protocol document was amended to reflect changes in protocol as a result of field experience.

### Field Audit Data Collection Protocol

The four cities protocol outlined three main elements of data collection: (1) data to collect, (2) methodology for data collection, and (3) organization of data into a consistent study format. Features identified for data collection efforts included on-street bicycle lanes, off-road paved trails for bicycles or pedestrian use, bicycle parking, parks, rail stations, and routes, and city bus routes (Table 1). Field audit data collection began in January 2012 and lasted through August 2012.

**Table 1:** **Sources of information for the four cities study field audit**.

Feature	Chicago, IL, USA	Minneapolis, MN, USA	Oakland, CA, USA	Birmingham, AL, USA
Bike parking	• Chicago Bike Program	• Paper Maps • University of Minnesota • Commuter Connection • Downtown Biking Guide • Metro Transit Website	• City of Oakland • Field Checks or Funding Sources	• Regional Planning Commission of Greater Birmingham (RPCGB) • Campus Planning Department of University of Alabama Birmingham • Personal contact
Bike lane and off-road trail	• Chicago Bike Program • Personal Contact	• Paper Maps • University of Minnesota	• City of Oakland • Personal Contact	• Regional Planning Commission of Greater Birmingham (RPCGB) • City of Birmingham • Campus Planning Department of University of Alabama Birmingham • Personal contact
Rail stations and routes	• Chicago Transit Authority • Metra Rail • Local Newspapers	• City of Minneapolis	• Alameda-Contra Costa Transit (AC Transit) • Bay Area Rapid Transit (BART)	
Bus routes	• Chicago Transit Authority • City of Chicago (GIS website)	• Paper Maps • City of Minneapolis • Twin Cities Metropolitan Area Transit GIS data	• Alameda-Contra Costa Transit (AC Transit) • Personal Contact • Paper Maps	• Birmingham-Jefferson County Transit Authority (BJCTA) • Paper Maps • Local Newspapers • Personal Contact
Parks	• City of Chicago (GIS Website) • Chicago Park District • Local Newspapers	• Minneapolis Park and Recreation Board	• City of Oakland • City of Piedmont • Personal Contact • Local Newspapers	• Campus Planning University of Alabama Birmingham • City of Birmingham • Birmingham Park and Recreation Board

Auditors first met with field representatives from local organizations, such as city planners and transportation engineers, to locate appropriate data. Auditors were instructed to collect data on the current status of each feature (e.g., bike lanes) and on feature changes of a substantial degree (e.g., changes in bike lanes) over the 25-year study period. For four cities definitions of feature changes of a substantial degree, see Table 2. Note that definitions of substantial changes vary by domain. Substantial changes in features were recorded, including data on the timing, location, and nature of change (e.g., number and length). A conservative approach was taken wherein all data on changes were collected with review by the study PIs and team to evaluate whether changes were indeed of a substantial degree.

**Table 2:** **Guidelines for determining whether a built environment feature changed to a substantial degree over the 25-year study period**.

Feature	Criteria for defining a substantial change in the feature
Bicycle lanes and on-road trails	During the time period of interest, was there a deliberate policy or effort to build additional lanes or on-road trails ≥300 ft in the area? Or whether the newly added one substantially increased the connectivity of the network (this also includes changes to regional connectivity)? If yes to either question, then it is a substantial change
Off-road paved trails	See on-road trails
Bicycle parking	During the time period of interest, was there a deliberate policy or effort to build additional bicycle parking with 3 or more racks? Has the supply of bicycle parking increased or decreased substantially (for example, has it more than doubled)? If most bicycle parking in town is provided “incidentally” and at the motivation of a developer or property manager, then it would not be a substantial change (unless all developers are doing it)
Parks	Have new parks been built, remodeled, or removed? Where and when? Parks need to be larger than neighborhood pocket parks. Has there been significant remodeling of parks? If so, when and where?
Rail stations	Have rail lines been extended or closed? New stations opened or old stations closed? Changes in service (later or earlier service, or weekend service cuts) would not be substantial changes. Extensions to others parts of town previously not served by rail would be considered a change of substantial degree
Rail routes	See rail stations
City bus routes	Was a line extended (or cut) by around 1 mile? Did the extended (or cut) route substantially change the connectivity of the network (this also includes changes to regional connectivity)? Service frequency was not considered as criteria

For each substantial change in a built environment feature, auditors were instructed to provide a GIS layer and data elements for an associated attribute table. GIS layers included standard metadata for each feature, including the projection, original data source and date, and units of scale (see Table 3). The attribute table design was specified in the data collection protocol to ensure compliance with the study’s coding convention. Each feature ID number began with an indicator for the city (2 = Birmingham, 3 = Chicago, 4 = Minneapolis, and 5 = Oakland), followed by a feature code (1–7 for, respectively, bicycle parking, bicycle lanes/on-road trails, rail stations, rail routes, city bus routes, parks, and off-road paved trails), followed by a 5-digit code indicating the specific feature (starting with 00001). For example, park #1 in Birmingham had a code of 2600001; bike lane #131 in Minneapolis had code 4200131. To the extent possible, we relied on written documentation about feature changes, such as city planning reports, physical maps, and GIS databases with changes in built environment over time. Local experts – individuals in the field with knowledge of the area and feature changes, such as city planning employees or members of advocacy organizations – were a key source of information when no written documentation existed. Auditors provided a qualitative indicator of the certainty of temporal estimate. Given the 25-year timespan of historic data collection, exact feature change dates could not always be confirmed, and auditors were asked to indicate whether time/date information was solid or based on an educated recollection of historic information. For all features, data fields comprised the dates of feature installations/openings/additions, remodeling, and closings, along with a dichotomous (0/1) temporal indicator of date certainty. Additional attributes varied by feature type (e.g., route extensions for trails and lanes, facility changes within parks, and numbers of bike racks at bike parking locations).

**Table 3:** **Attribute data collected for each feature change of a substantial degree**.

Feature	Attribute data
For all features	• For each feature, the year it opened/added, remodeled, and/or closed. Text fields for notes related to data quality (e.g., uncertainty about dates)
Bicycle parking	• Number of parking racks every year • Number of parking spaces every year
Bicycle lanes and on-road trails	• Year when the trail extends outside of the city boundary
Rail stations and routes	• Year when the rail route extends outside of the city boundary
City bus routes	• Year when the bus route extends outside of the city boundary
Parks	• Name of park • Sports fields (year of substantial changes) • Sports fields are defined as fields set aside for sports including courts, baseball diamonds, soccer fields, frisbee golf, etc. Plain green space was not considered to be a sports field • Swimming pools (year of substantial changes) • Community center (year of substantial changes) • Type of park (codes for: pocket park, neighborhood park, city park, regional park, and unknown) • Pay facility (year of substantial changes). If some of the park facilities are pay (for example, pools) and some are unpaid (playgrounds), please consider it as a pay facility • Trails in parks should be recorded as an off-road paved trail (see next item)
Off-road paved trails	• Name of trail • Material (codes for: pavement, concrete, other, and unknown) • Year when the trail extends outside of the city boundary

### Field Audit Data Verification with CIP, TIP, and PCIP

An independent set of team members was selected based on experience and professional training in city/regional planning and/or transportation engineering to verify and expand on the on-the-ground audit. We approached the verification process of the field audit data using transportation improvement program (TIP) plans, capital improvement plans (CIP), and park capital improvement program (PCIP) plans for each of the cities obtained by contacting the city public works, metropolitan planning organization, and/or parks departments for each city. These plans helped to provide verification, including dates or timetable of investment, size or cost of investment, specific features modified during the investment, and programmed changes in features. CIPs and PCIPs provided data on recreational investments *via* short-range investment plans, usually 4–6 years, which identified capital projects and equipment purchases, a timetable and identified financing options, and a link between a municipality, school district, parks and recreation department and/or other local government entity, and a comprehensive and strategic plan and the entity’s annual budget (e.g., http://web.archive.org/web/20120308142650/http:/www.metrocouncil.org/parks/CIP.pdf). The TIPs provided data on programmed changes for bike lanes, bike racks, off-road trails, and rail stations. TIPs are public documents, required by Federal Law, that summarize the proposed transportation investments for the following 5–7 years, categorized by type and source of funds. TIPs were updated annually in the 1990s and every 2 years post 1998. Although estimated completion dates are given for projects in the plan, it should be noted that the TIP is not a CIP. Unlike CIPs, TIPs represent an agency’s intent to construct or implement a specific project and the anticipated flow of federal funds and matching state or local contributions (57). In cases where CIP and TIP documents were not available, CIP and TIP documents from the year prior were used as appropriate. For example, Minneapolis did not have a 1988 CIP, but the 1987 CIP had information on projects for parks from 1988, thus providing verification details. In this case, 1987 CIP document is used to verify 1988 projects for parks. It is important to note that not all projects would be registered in TIP, CIP, or PCIPs. Those projects may be programmed and funded through other mechanisms, such as regular operating budgets, school budgets, and bond proceeds. For this reason, the verification was considered exploratory, in the sense that it provided information about the policy landscape in each location but limited the possibility of conducting a formal validation.

Several rules were applied for the verification process as follows: the project completion year was defined as the change/open year. If the completion year was unavailable, then the commencement year was used as the change/open year. Updates were restricted to projects valued at $1,000 or more and projects that affect physical accessibility to the feature. Thus, projects such as fencing or lighting in parks were not included. We also calculated the annual investment amount for the four cities based on CIPs and TIPs, using different approaches based on data available for each city. For Chicago, Minneapolis, and Oakland, annual investment amounts were calculated as the aggregate investment for each project. For Birmingham, the annual investment amount was obtained from the yearly total investment amount table provided in the TIP and CIP documents.

For bicycle lanes and off-road trails, a brief examination of the files and consultation with local planners enabled the research team to identify these features using different types/names (e.g., greenway, rail-trail way, and multi-use trails) and make decisions regarding whether the specific feature served as bike or off-road trails. Then, the researcher used Google Maps to locate the lanes based on the description of the start and the end points of the change in the GIS field audit file. For parks, researchers identified the park name, project description (e.g., opening, addition, modeling and closing of sports field, swimming pool, or community center) and the year of the change from the CIPs or PCIPs file. If a feature already existed in the GIS field audit file, the verifier checked to see if the change noted in the CIPs or TIPs had been recorded. Changes were recorded if they were not present in the GIS field audit file. All of the cities experienced changes in their bicycle lanes and off-road trails and all excluding Chicago also saw additions of new lanes and trails. Chicago and Oakland made additions to their parks, and all cities excluding Birmingham made changes to their parks. Only Chicago and Minneapolis experienced a change in rail network during the time period studied.

### Data Cleaning and Processing

All data were cleaned and harmonized across all four cities by the verification team using ArcGIS (ESRI, Redlands, CA, USA). Once delivered to study team personnel at UNC, the field audit data were verified and further standardized across all four cities. This data harmonization process and preliminary data analysis occasionally revealed the need for additional data corrections. For example, the recreational trail and bike lane data were modified to address topological inconsistencies, such as unintended small gaps along linear segments. Finalized geospatial datasets were subsequently processed in ArcGIS by a spatial technician from the Spatial Analysis Unit of the Carolina Population Center at UNC to aggregate measures to higher geographic levels (e.g., neighborhoods, tract, and zip code) and integrate measures with CARDIA participant data from the clinic visits at each exam.

Neighborhood features were compiled for each geographic level and every year (Table S1 in Supplementary Material) using a set of fixed neighborhood boundaries to isolate changes in features from simple boundary changes. Features and resources within 23-m (75-ft, determined by average road width) of a neighborhood were attributed to that neighborhood. Along a boundary shared between two neighborhoods, features within 23-m of each boundary were included in the data measures for both to reflect amenities that could be easily accessed by each neighborhood. This is because a bike lane, for example, serves both neighborhoods even if a street is between the neighborhood and the bike lane.

Estimates of features and resources were linked to each individual CARDIA participant’s geocoded address at every exam year. The geocoded residential location of a respondent is available for as many as six time points: circa 1985–1986, 1992–1993, 1995–1996, 2000–2001, 2005–2006, and 2010–2011 (corresponding to CARDIA exam years 0, 7, 10, 15, 20, and 25, respectively). For any exam period, a respondent’s location may be within the four cities, outside the four cities, or unknown (e.g., absent from the study). Field audit data measures were assigned to individual respondents for each year that they lived in the four cities. Multi-year spans of audit data were assigned to each participant location by calculating the mid-point years between CARDIA exams and assuming that the participant remained in the same residence during that time (preceding inter-exam midpoint to succeeding inter-exam midpoint). Since 75% of participants remain in these cities, we have a rich longitudinal dataset of these individuals.

The proximity and accessibility of audited transportation infrastructure and recreational facilities relative to each participant’s residential location were measured in multiple ways, as appropriate for each feature type (Table S1 in Supplementary Material). Distance to the nearest available feature was calculated in both Euclidean and network distance. Where relevant, other measures represent features within 250-m, 500-m, 1-km, 3-km, 5-km, and 8.05-km Euclidean and network buffers around the respondent’s geocoded residential location. For parks, we also calculated an inverse-distance weighted accessibility index to all parks in each city. Where a participant’s buffer extended beyond the boundaries of data, an indicator of the area within the city boundaries was created for use in analyses.

## Results

Not all CIP, TIP, and PCIP documents were available at all periods (see Table 4). Birmingham was especially challenging to gather data from, with no TIP until 1993 and no CIP or PCIP. In general, rail, trail, and park data were easier to gather from these documents than bicycle data.

**Table 4:** **Availability of CIP, PCIP, and TIP by year for verification of spatial elements and feature tables**.

	Chicago, IL, USA			Birmingham, AL, USA^a^	Oakland, CA, USA		Minneapolis, MN, USA	
Year	CIP	TIP	PCIP	TIP	CIP	TIP	CIP	TIP
1985	P, R	A			P, T	A	T	A
1986	P, R				B, P, T	A	B, P, T	T

1987	R					A	B, P	T
1988	P, R	A			P	A		T

1989						A	A	A
1990	R					A	A	A
1991					P	A	P, T	A

1992	R	A				A	P, T	A
1993	R		A	A	P	A	P, T	A
1994		A		A		A	P, T	T

1995			A	A	P	A	P, T	T
1996	R				P		P, T	T
1997		A	A		P, T	A	P, T	A
1998			A				P, T	T
1999				A		A	P, T	T

2000	R, T		P				P, T	T
2001		R	P	T	B, P, T		T	T
2002				A			T	A
2003	R, T		P				T	A
2004	T		P	A			T	T

2005	T		P				B, T	T
2006			A	A			B, T	T
2007		R	A		P, T	B, T	T	B, T
2008	R, T		P	A			T	T
2009			P			B, T	P, T	B, T

2010	A	A	P				P, T	B, T
2011	A		P				A	B, T
2012	A		A				A	T

*^a^CIP was not available for Birmingham, AL, USA*.

*A: Data available, but no description regarding the spatial elements of interest*.

*B: Bike lanes available*.

*P: Parks available*.

*R: Rails available*.

*T: Trails available*.

**Figure 2:**
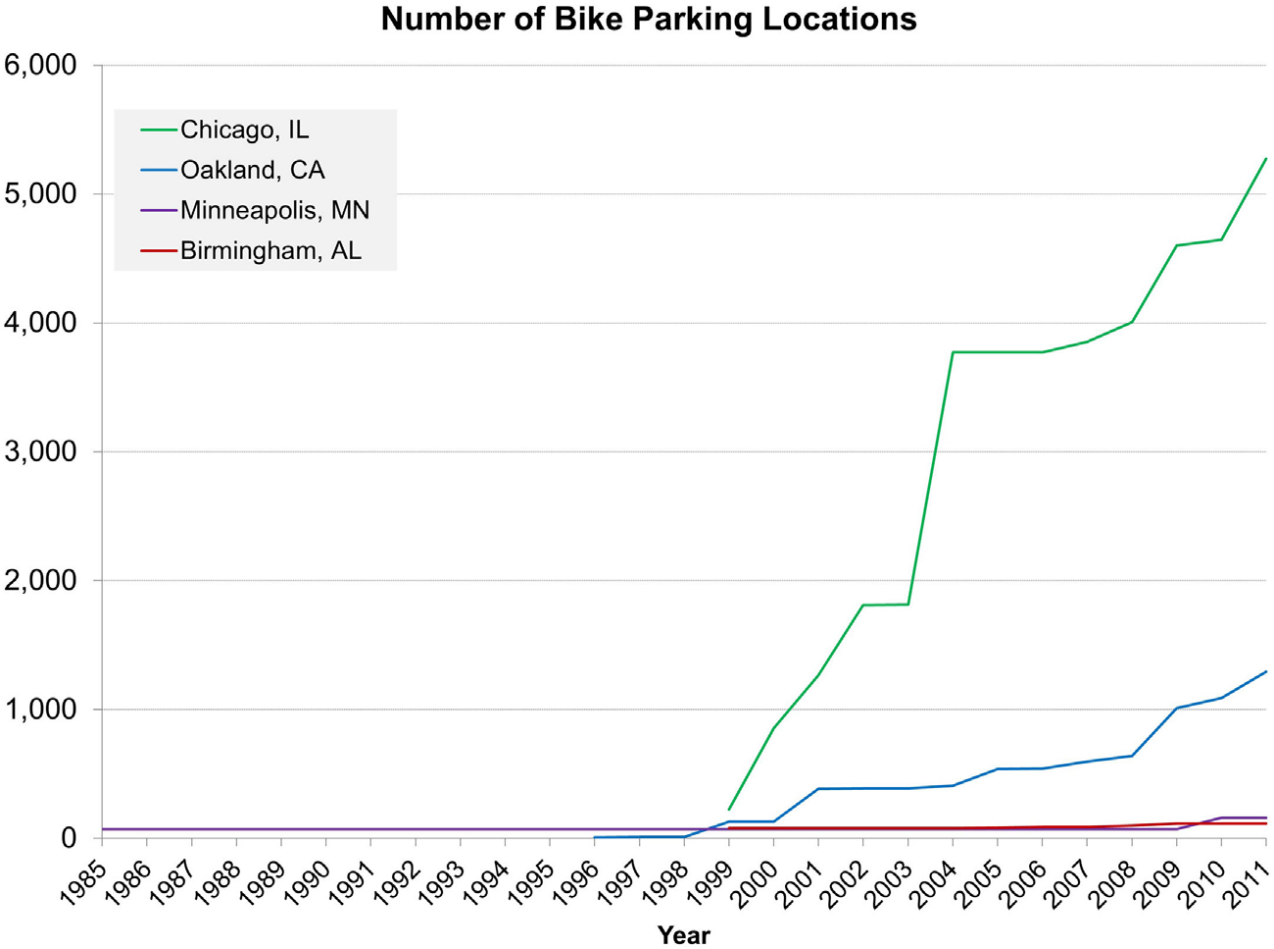
**Change in number of bicycle parking locations between 1985 and 2011 by city in the CARDIA four cities field audit**. Green represents Chicago, IL, USA; blue represents Oakland, CA, USA; purple represents Minneapolis, MN, USA; red represents Birmingham, AL, USA.

**Figure 3:**
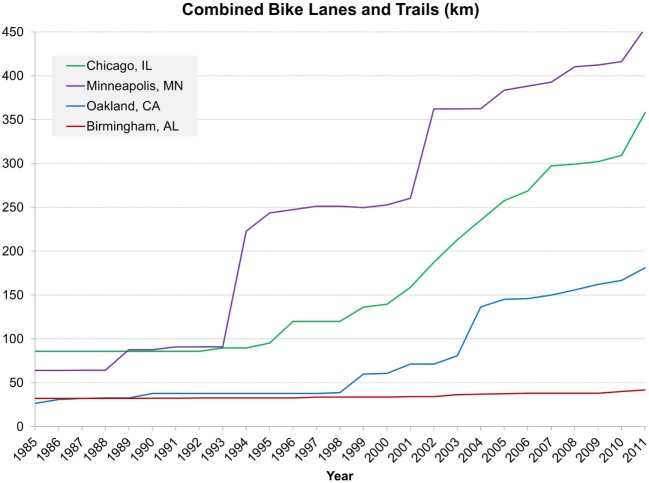
**Change in distance of bicycle lanes and recreational trails (kilometers) between 1985 and 2011 by city in the CARDIA four cities field audit**. Green represents Chicago, IL, USA; blue represents Oakland, CA, USA; purple represents Minneapolis, MN, USA; red represents Birmingham, AL, USA.

Bicycle parking, lanes, and recreation trails all increased dramatically between 1985 and 2011. Most notably, bicycle parking increased in Chicago, IL and Oakland, CA (USA) starting in 2000 (Figure 2). Similarly, tremendous growth occurred in bicycle lanes and recreational trails in all cities except Birmingham, AL, USA starting in the mid 1990s and continuing until the end of the retrospective audit (Figure 3). Minneapolis, MN, USA experienced the largest increase, followed by Chicago, IL, USA, and then by Oakland, CA, USA. During the duration of the retrospective audit, minimal changes occurred in bus service, rail lines and stations, and parks (Figures S6–S9 in Supplementary Material). However, bus service increased slightly in Birmingham, AL and Minneapolis, MN (USA). In addition, while Minneapolis, MN and Birmingham, AL (USA) did not have rail lines for the earlier part of the study, Minneapolis, MN, USA started a rail line in 2004 with 10 stations (adding 1 more station post-completion).

## Discussion

This protocol details methods to collect, verify, and process historic data on the built environment in the field audit component of the CARDIA four cities study. We developed and verified retrospective field audits in four cities [Birmingham, AL; Chicago, IL; Minneapolis, MN; and Oakland, CA (USA)] to capture introductions, renovations, and closures representing changes in recreation facilities (e.g., trails and parks) and transportation infrastructure (e.g., bus, light rail, bike parking, and bike paths). These derived data were then linked to CARDIA sites’ respondents’ geographic locations over the 25-year study period to examine associations with health behaviors and outcomes. Our methodology can be used as a model for other studies seeking to generate detailed data on historical changes in built environment features over a long period of time.

Several practical and operational issues arose during these retrospective audits. One of the primary practical issues surrounded the level of detail for data collection, which required balance between parsimony and level of detail. The first major issue was determining what changes in features represented a substantial change. For example, we discussed situations regarding extensions of bus routes for specified distances to determine a cut point that would represent a substantial change in bus service for a given location. Similarly, we discussed whether a specified number of new bicycle parking spaces would be considered a change of a substantial degree. The guidelines specified in this paper demonstrate our balance between parsimony and sufficient data to capture changes in features. Although we present our guidelines for determining substantial change, some decisions required case-by-case attention given differences in detailed reporting of changes from various sources across cities. Additionally, this data collection was limited by a focus on infrastructure features (i.e., bicycle, trail, park, and transit). Thus, this protocol does not outline methods for capturing larger-scale built environment characteristics (e.g., density and land use mix).

Generally, cities are not collecting data for research, and it is the responsibility of researchers to forge partnerships that enable archiving of data for longitudinal and historic research. There was substantial variability in historic data and documentation by city. Some attributes were particularly challenging to document. Bicycle parking was one such feature due to the nature of reporting by local city governments, which was more focused on *status quo* rather than changes. In some cases, local officials and municipal employees overwrote or deleted data that they deemed “old” and replaced data with updated information. Additionally, verification was unequal across cities due to differences in availability of CIPs, TIPs, and PCIPs. This limitation might result in differential percentages of data verified by city or more accurate data for one city over another. Another contributing factor was unevenness in willingness to provide access to data. This is likely due to differences in the allocation of resources devoted to these issues or the internal structure of departments within cities. It is critical for researchers to create partnerships with local governments in order to archive data for future research.

Our choice to include auditors and verifiers with intimate knowledge of the cities was critical for decision-making around apparent inconsistencies in the data. While some auditors had knowledge of, and documented information about, specific people or sources of information, others had to rely solely on the information gleaned from the retrospective audit. For example, in several of our cities, we had names and information for key contacts, whereas in others, we only had the primary data source for each data element. Local, non-institutional community organizations also played a key role. For example, in Birmingham, we were able to meet a citizen who had collected all transit maps. Creating partnerships with local groups can also represent a bi-directional endeavor that can increase communication between researchers and communities; in Oakland, CA, USA, we worked with a group of city planners who were interested in seeing results and aggregated information after completion of data collection for their own purposes. Thus, there was clear benefit of our data collection for these local organizations.

Despite these complexities, our method provides a novel and valuable direction in the collection of retrospective neighborhood data for use in longitudinal analyses. Although the data produced have limitations that would not exist with prospectively collected data, they give insight into the way neighborhoods have changed over time. Our ability to link these data to the CARDIA clinical exam data allows researchers the potential for studies assessing the relationship between built environment changes and health behaviors. Additionally, the process of performing these field audits builds a network of informants within research cities that can help fuel new research questions, built evidence around appropriate model specifications, interpret research findings, and ultimately bridge the gap between research and cities to translate findings back into meaningful neighborhood policies. Given the importance of these data for research and the valuable direct application to resource allocation, future work should strive to build relationships with local governments around data collection and management.

## Conclusion

This methodology details the innovative process of performing retrospective field audits of built environment data. Although the data produced have limitations that would not exist with prospectively collected data, they give insight into the way neighborhoods have changed over time. Specifically, we found large increases in bicycle infrastructure. Our methodology can be used as a model for other studies seeking to generate detailed data on historical changes in built environment features over a long period of time. Measures from this data compilation process can be combined with existing longitudinal cohort studies to examine critical questions of neighborhood resources’ influence on health behaviors and health outcomes.

## Author Notes

JH is a postdoctoral fellow in the Carolina Population Center at the University of North Carolina at Chapel Hill. She holds a PhD in Epidemiologic Science from the University of Michigan, a Master in Environmental Studies, and a Bachelor of Arts in Environmental Studies, Health and Societies and Nutrition from the University of Pennsylvania. KM is a Research Assistant Professor in the Nutrition Department at the University of North Carolina at Chapel Hill. She holds a ScD in Epidemiology from Harvard University, a Master of Public Health in Epidemiology from the University of Minnesota, and a Bachelor of Arts in Art History from Macalester College. MP is a Research Associate and Senior Spatial Analyst at the Carolina Population Center. He has a Master of Arts in Geography and Bachelor of Arts in Earth Science, both from the University of Northern Iowa. He has 15 years of geospatial and satellite remote sensing analytic and modeling experience. DR is a Distinguished Professor of Sustainable Community Design in the Department of City and Regional Planning, an Adjunct Professor in the Department of Epidemiology, and the Director of the Center for Sustainable Community Design within the Institute for the Environment at the University of North Carolina at Chapel Hill. He holds a PhD in Urban Planning from the University of Michigan, a Master of Science in Transportation from the Massachusetts Institute of Technology, and a Bachelor of Science from Fordham University. YS is a professor in the Department of City and Regional Planning at the University of North Carolina at Chapel Hill. She holds a PhD in City and Regional Planning from the University of Illinois at Urbana-Champaign. KP is a doctoral student in the Department of City and Regional Planning at the University of North Carolina at Chapel Hill. She holds a Master of City and Regional Planning from the University of North Carolina at Chapel Hill, a Master of Science in Architecture Design and Theory from Shenzhen University, and a Bachelor of Arts in Architecture Design and Theory from Hunan University. JH is a doctoral student in the Department of City and Regional Planning at the University of North Carolina at Chapel Hill. He holds a Bachelor of Science and Master of Science in Civil and Environmental Engineering from Sung Kyun Kwan University and a Master of City and Regional Planning from the University of North Carolina at Chapel Hill. PG-L is a Professor in the Department of Nutrition and the Carolina Population Center at the University of North Carolina at Chapel Hill. She holds a PhD and Master of Arts in Physical Anthropology from the University of Pennsylvania and a Bachelor of Arts in Anthropology and Experimental Psychology from Tulane University.

## Author Contributions

JH and KM drafted the study protocol manuscript. All authors reviewed and contributed to the final draft. PG-L, DR, MP, and YS were involved in data generation and analysis. JH, KP, and MP were involved in GIS processing. PG-L secured funding to perform the ancillary study.

## Conflict of Interest Statement

The authors declare that the research was conducted in the absence of any commercial or financial relationships that could be construed as a potential conflict of interest.

## Abbreviations

CARDIA, coronary artery risk development in young adults; CIP, capital improvement plan; GIS, geographic information systems; PCIP, park capital improvement program; TIP, transportation improvement plan; UNC, University of North Carolina.
