# Supplementary material for: Obtaining Longitudinal Built Environment Data Retrospectively across 25 years in Four US Cities
Source: Front Public Health. 2016 Apr 19;4:65. doi: 10.3389/fpubh.2016.00065 (PMC4835448; doi:10.3389/fpubh.2016.00065)

SUPPLEMENTAL TABLE:

**Table S1: Constructed variables at the neighborhood and individual level from the Four Cities field audit data.**		
**Level**	**Constructed Variables**	**Original Source Data**
Neighborhood	Number of bicycle parking locations within each neighborhood.	Bicycle parking points
Neighborhood	Number of bicycle parking racks within each neighborhood.	Bicycle parking points
Neighborhood	The mean distance to the nearest bike parking location from sample points spaced every 10 meters throughout the neighborhood.	Bicycle parking points
Neighborhood	Total length of bike lane or trail features within each neighborhood.	Bicycle lane and/or trail lines
Neighborhood	Bicycle and/or trail connectivity: number of discrete networks, unconnected to each other	Bicycle lane and/or trail lines
Neighborhood	Bicycle and/or trail connectivity: number of linear segments	Bicycle lane and/or trail lines
Neighborhood	Bicycle and/or trail connectivity: number of network intersections	Bicycle lane and/or trail lines
Neighborhood	Bicycle and/or trail connectivity: number of end points (bike lane or trail terminus point)	Bicycle lane and/or trail lines
Neighborhood	Bicycle and/or trail connectivity: number of 3-way intersections (‘T’ or’ Y’ intersections)	Bicycle lane and/or trail lines
Neighborhood	Bicycle and/or trail connectivity: number of 4-way intersections	Bicycle lane and/or trail lines
Neighborhood	Bicycle and/or trail connectivity: number of 5-way intersections	Bicycle lane and/or trail lines
Neighborhood	Bicycle and/or trail connectivity[a]: network cyclomatic index: L–V+1	Bicycle lane and/or trail lines
Neighborhood	Bicycle and/or trail connectivity[a]: network alpha index: CYCLO / (2V–5)	Bicycle lane and/or trail lines
Neighborhood	Bicycle and/or trail connectivity[a]: network beta index: L/V	Bicycle lane and/or trail lines
Neighborhood	Bicycle and/or trail connectivity[a]: network gamma index: L / (3(V–2))	Bicycle lane and/or trail lines
Neighborhood	Bicycle and/or trail connectivity[a]: intersection proportion: (V−J1)/V	Bicycle lane and/or trail lines
Neighborhood	The mean distance to the nearest of bike, trail, or unified bike and trail features from sample points spaced every 10 meters throughout the neighborhood.	Bicycle lane and/or trail lines
Neighborhood	Total length of bike lane, trail, or unified bike lane and trail features that may be traversed starting from access points within the neighborhood. In areas with extensive networks, the full reach may span tens of kilometers from the neighborhood.	Bicycle lane and/or trail lines
Neighborhood	Total length of streets with bus service within each neighborhood.	Bus service lines
Neighborhood	Proportion of total street length within the neighborhood which has bus transit service.	Bus service lines
Neighborhood	The mean distance to the nearest street with bus service from sample points spaced every 10 meters throughout the neighborhood.	Bus service lines
Neighborhood	Number of rail stations within each neighborhood.	Local rail lines
Neighborhood	The mean distance to the nearest rail station from sample points spaced every 10 meters throughout the neighborhood.	Local rail lines
Neighborhood	Number of positive significant station changes within the neighborhood since the previous year	Local rail lines
Neighborhood	Sum length of rail lines within each neighborhood.	Local rail lines
Neighborhood	The total length—in each year—of the entire rail system which serves the city	Local rail lines
Neighborhood	Count of parks accessible within the neighborhood	Park polygons
Neighborhood	Count of parks served by on-street bicycle lane features	Park polygons
Neighborhood	Count of parks served by recreational trail features	Park polygons

Neighborhood	Count of parks per neighborhood with community centers	Park polygons
Neighborhood	Count of parks per neighborhood with swimming pools	Park polygons
Neighborhood	Count of parks per neighborhood with sports fields	Park polygons
Neighborhood	Number of positive significant park changes within the neighborhood since the previous year	Park polygons
Neighborhood	The mean distance to the nearest park from sample points spaced every 10 meters throughout the neighborhood. Sample points within a park have distance=0.	Park polygons
Neighborhood	Total park area of all parks accessible from the neighborhood.	Park polygons
Neighborhood	Total park area within each neighborhood.	Park polygons
Neighborhood	Total park area of parks accessible from the neighborhood, only counting parks which have one or more facilities: bike lane, trail, community center, swimming pool, or sports field.	Park polygons
Individual	Euclidean and network distances from each participant residence location to the nearest bicycle parking location.	Bicycle parking points
Individual	Number of bicycle parking locations within each Euclidean and network buffer distance from the respondent's residential location.	Bicycle parking points
Individual	Number of bicycle parking racks within each buffer distance.	Bicycle parking points
Individual	Weighted count of bicycle parking locations within each Euclidean and network buffer distance.	Bicycle parking points
Individual	Bicycle Parking Index within each buffer distance: the sum of the weighted count of each bicycle parking location (see above) multiplied by the number of racks.	Bicycle parking points
Individual	Euclidean and network distances from each participant location to the nearest bike lane feature and nearest trail feature	Bike lane and/or trail lines
Individual	Total length of bike lane or trail features within each Euclidean and network buffer distance.	Bike lane and/or trail lines
Individual	Connectivity measures for bike lanes and trails within each Euclidean buffer distance.	Bike lane and/or trail lines
Individual	Total length of bike lane, trail, or unified bike lane and trail features that may be traversed starting from access points within a respondent's buffer.	Bike lane and/or trail lines
Individual	Euclidean and network distances from each participant residential location to the nearest street with bus transit service.	Bus service lines
Individual	Total length of streets with bus service within each buffer distance from the respondent's residential location.	Bus service lines
Individual	Proportion of total street length within the buffer which has bus transit service.	Bus service lines
Individual	Euclidean and network distances from each participant residence location to the nearest commuter rail station	Local rail station points
Individual	Euclidean distance from each participant residence location to the nearest location along a commuter rail line.	Local rail lines
Individual	Number of rail stations within each Euclidean and network buffer distance from the respondent's residential location.	Local rail station points
Individual	Number of positive significant station changes within the buffer since the previous year (mostly refurbishings; negative changes are closures, reflected in the count).	Local rail station points
Individual	Total length of commuter rail lines within each respondent buffer.	Local rail lines
Individual	Euclidean and network distances from each participant residence location to the nearest park of any size and the nearest magnet park.	Park polygons
Individual	Park accessibility index 1: an inverse distance weighted score (i.e., weighted count) of parks accessible within each search distance. Parks within 400 m of the respondent location are counted with full weight = 1 and for each park farther than 400 m, that park's contribution to the index diminishes with increasing distance.	Park polygons

Individual	Park accessibility index 2: similar to index 1 except that the count weight of each park is multiplied by the park area in square km. The contribution of each park to the index total increases as its land area increases and this weight may amplify or counteract the inverse-distance weight.	Park polygons
Individual	Counts of parks and magnet parks accessible within the respondent buffer.	Park polygons
Individual	Count of parks per buffer with sports fields	Park polygons
Individual	Count of parks per buffer with swimming pools	Park polygons
Individual	Count of parks per buffer with community centers	Park polygons
Individual	Count of parks served by bicycle lanes in our field audit database	Park polygons
Individual	Count of parks served by off-road trails in our field audit database	Park polygons
Individual	Number of positive significant park changes within the respondent buffer since the previous year (mostly refurbishings; most negative changes are closures, which will be reflected in the above counts).	Park polygons
Individual	Total park area (m2) of all parks accessible from each respondent buffer.	Park polygons
Individual	Total park area (m2) within the respondent buffer.	Park polygons
Individual	Total park area (m2) of parks accessible from each respondent buffer and with one or more audited facilities present (sports field, swimming pool, community center, bike lane, or trail).	Park polygons
Individual	Total park area (m2) of all parks accessible from each respondent buffer.	Park polygons

[a]For connectivity equations, L represents the number of links (connections between nodes), V represents the number of nodes (intersections), J1 represents the number of single-valent nodes (cul-de-sacs or dead end streets), and J*n* represents the number of n-way intersections (i.e. J3 is an intersection with three links meeting at the node, J4 is 4-way intersection, etc.). The formulas are based on classical graph theory, in which networks are closed and every link has a node at each end. As a result of identifying links and intersections within externally imposed boundaries or limits, however, some links have one end inside the measurement area and one node omitted from the calculation. This decrease in the number of nodes vis-à-vis a closed network produces values outside the theoretical ranges, but which still fall along a meaningful continuum.

SUPPLEMENTAL FIGURES:

Figure S1: Bicycle parking locations present during any year throughout the 1985 to 2011 survey period. Purple dots represent each bicycle parking location. All years overlaid.

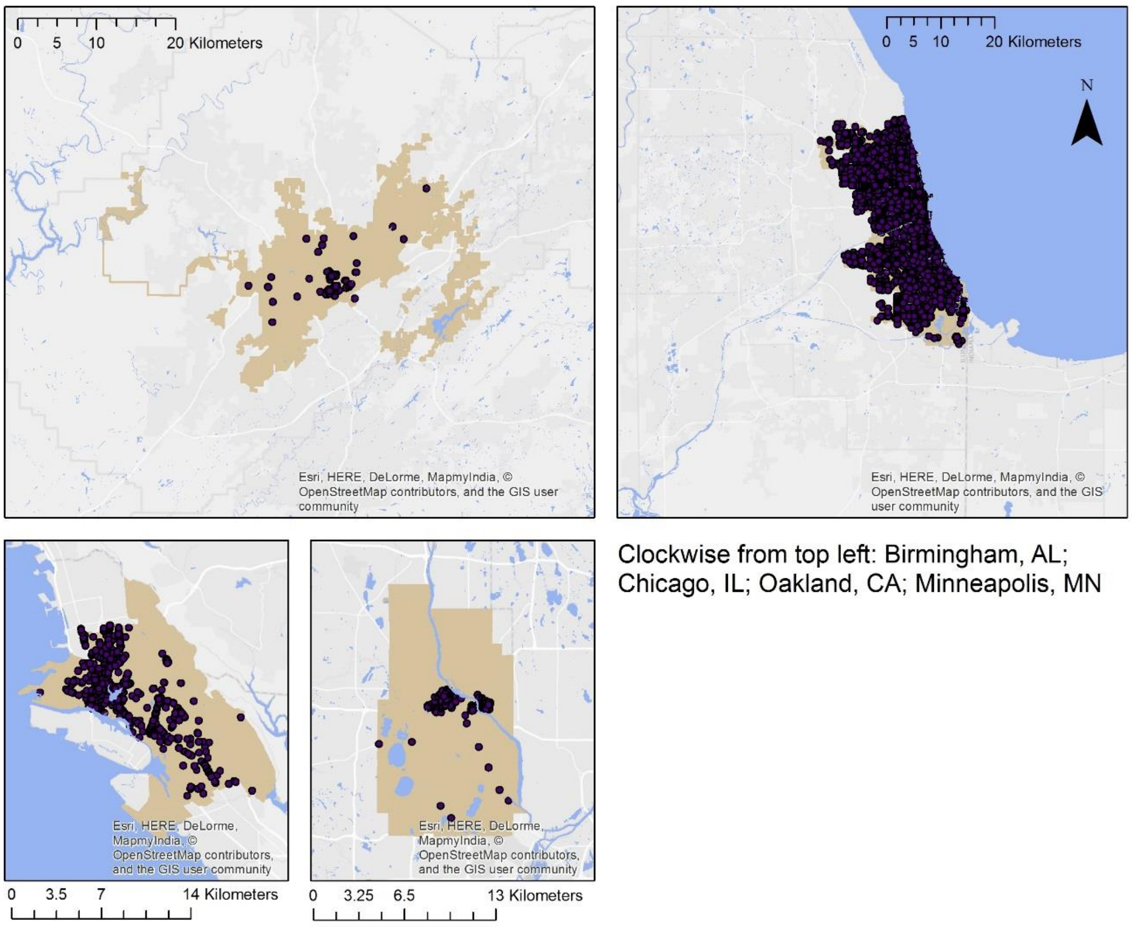

Figure S2: Bicycle lanes and recreational trails present during any year throughout the 1985 to 2011 survey period. Purple lines represent each lane or trail. All years overlaid.

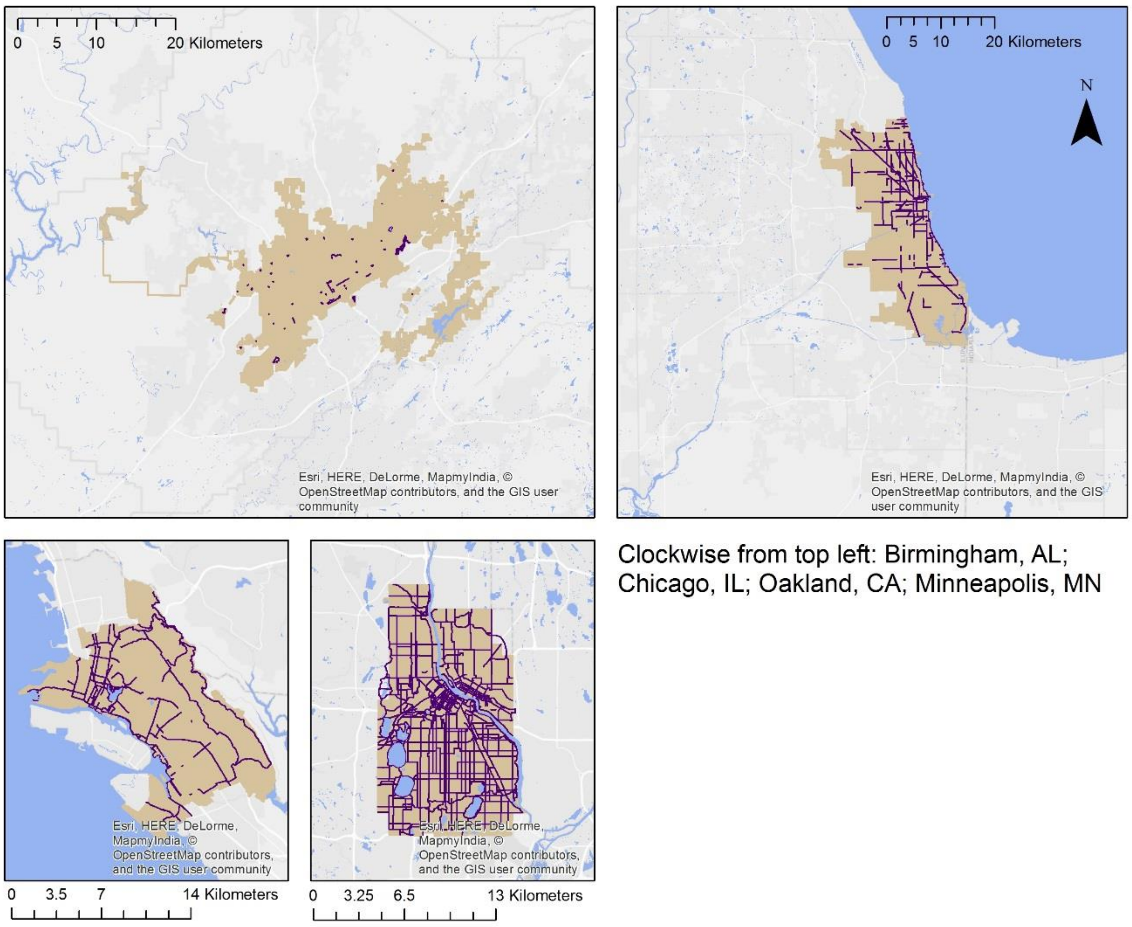

Figure S3: Bus service present during any year throughout the 1985 to 2011 survey period. Red lines indicate bus service. All years overlaid.

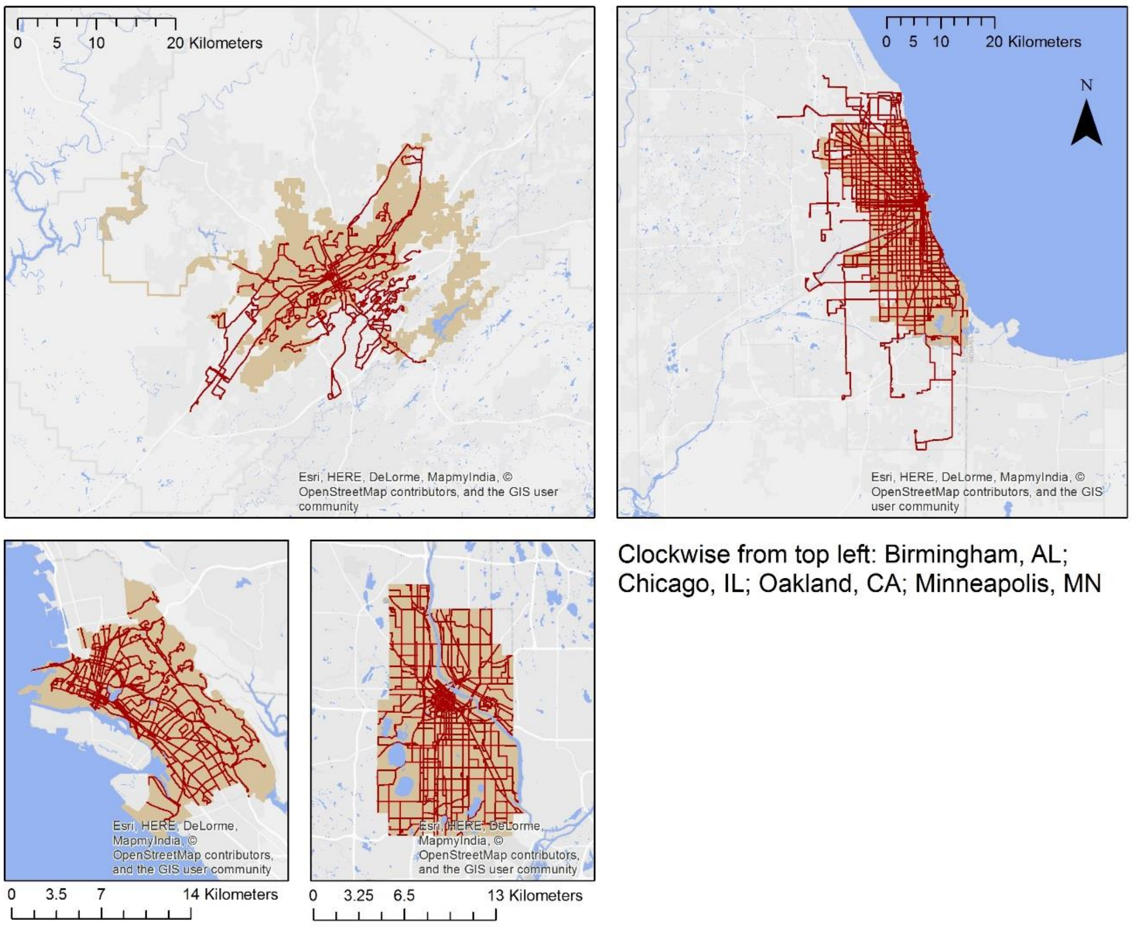

Figure S4: Rail stations and lines present during any year throughout the 1985 to 2011 survey period. Blue dots represent stations and blue lines represent lines. All years overlaid. No rail service in Birmingham, AL.

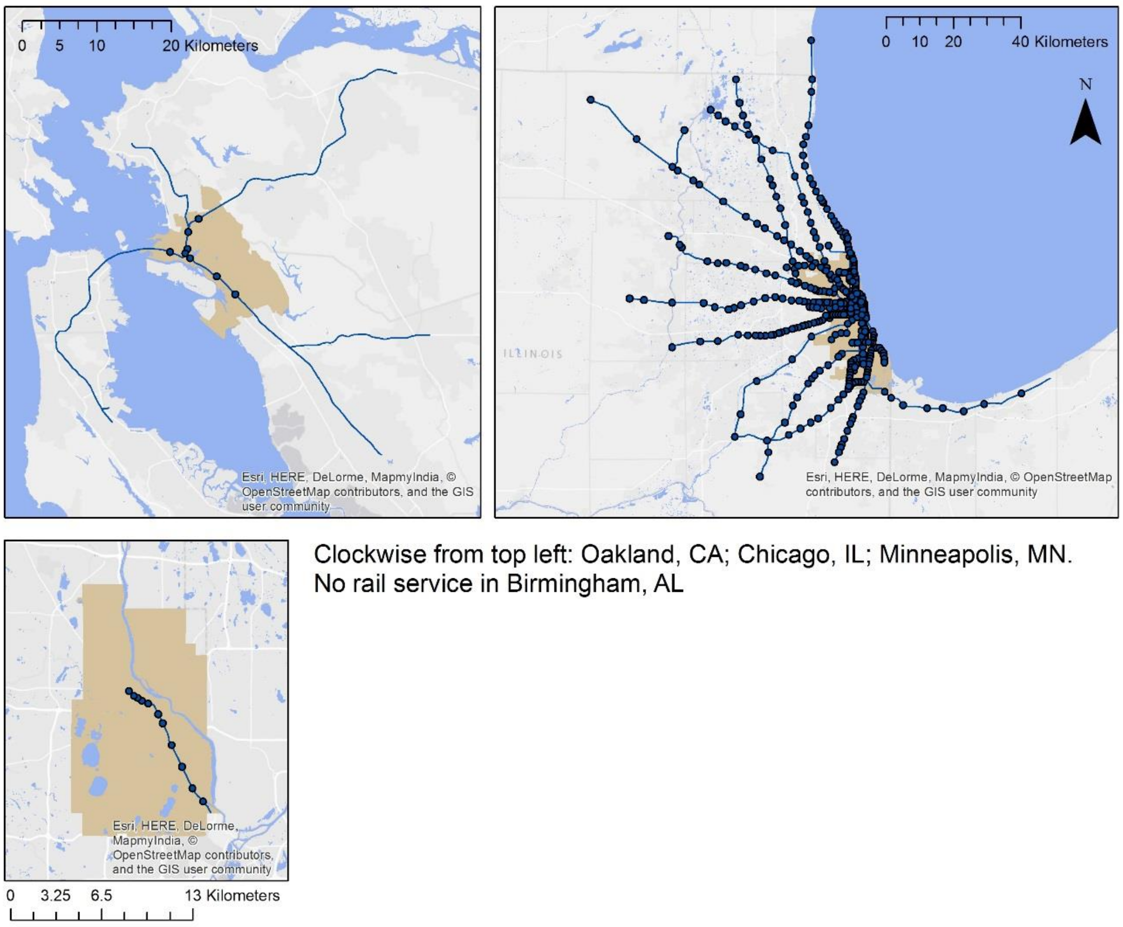

Figure S5: Parks present during any year throughout the 1985 to 2011 survey period. Green area represents each park polygon. All years overlaid.

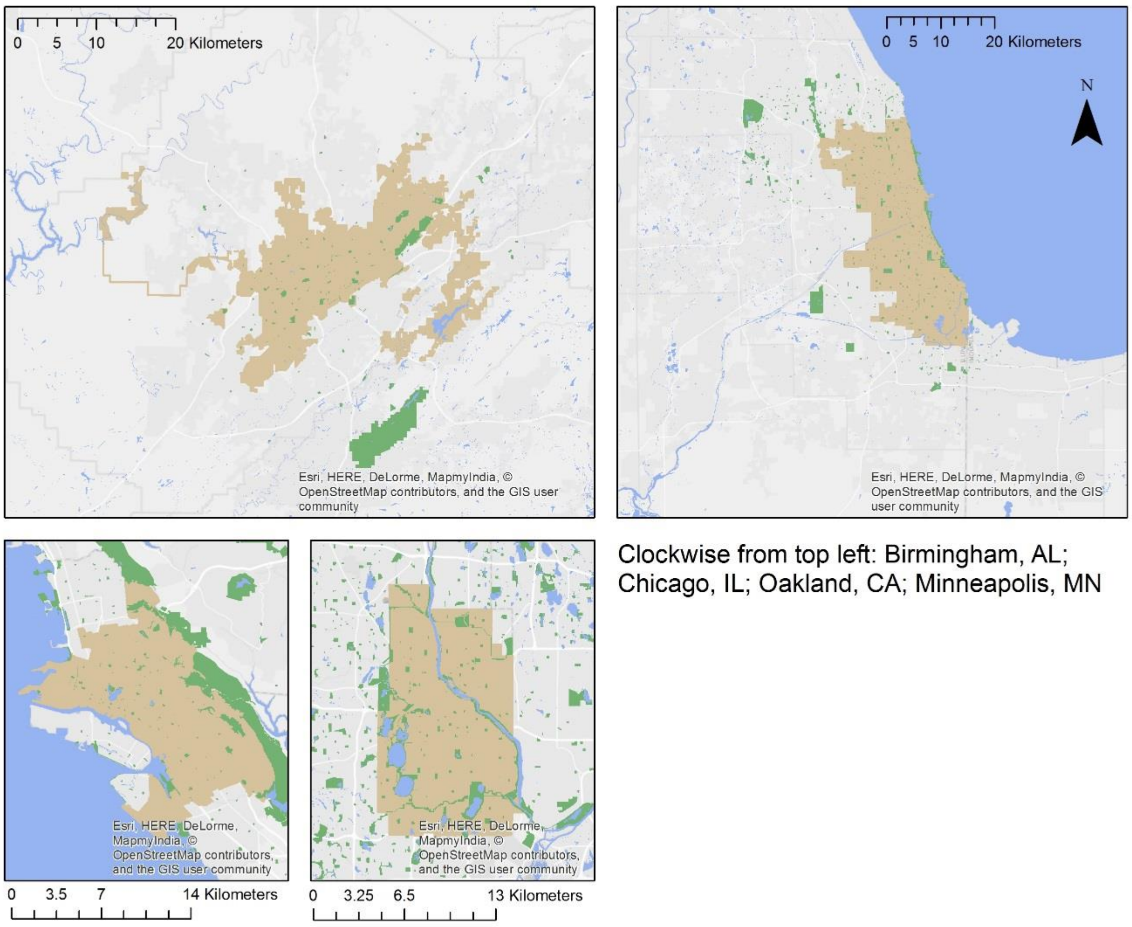

Figure S6: Change in streets with bus service (km) between 1985 and 2011 by city in the CARDIA Four Cities field audit. Green represents Chicago, IL; blue represents Oakland, CA; purple represents Minneapolis, MN; red represents Birmingham, AL.

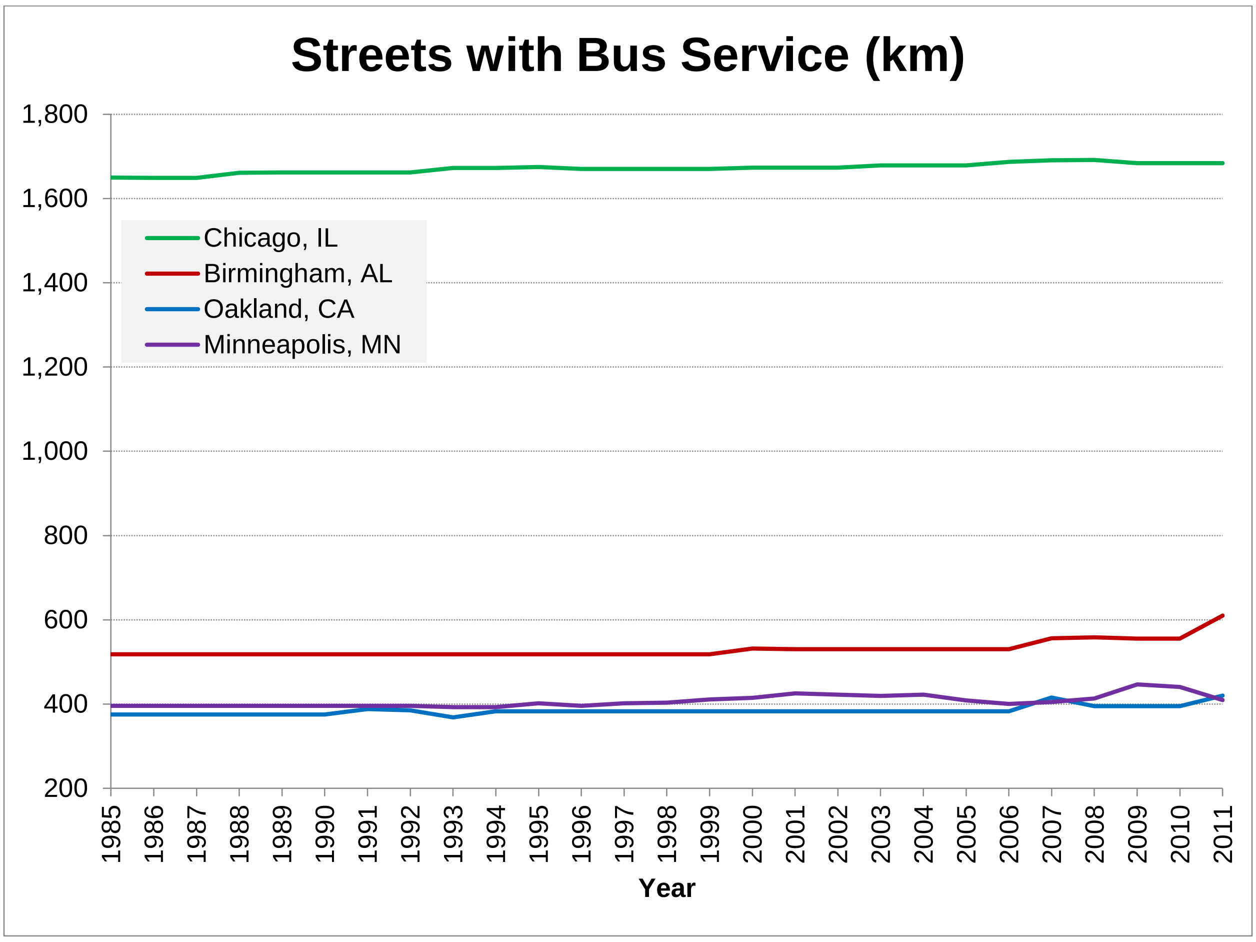

Figure S7: Change in number of rail stations between 1985 and 2011 by city in the CARDIA Four Cities field audit. No rail in Birmingham, AL. Green represents Chicago, IL; blue represents Oakland, CA; purple represents Minneapolis, MN; red represents Birmingham, AL.

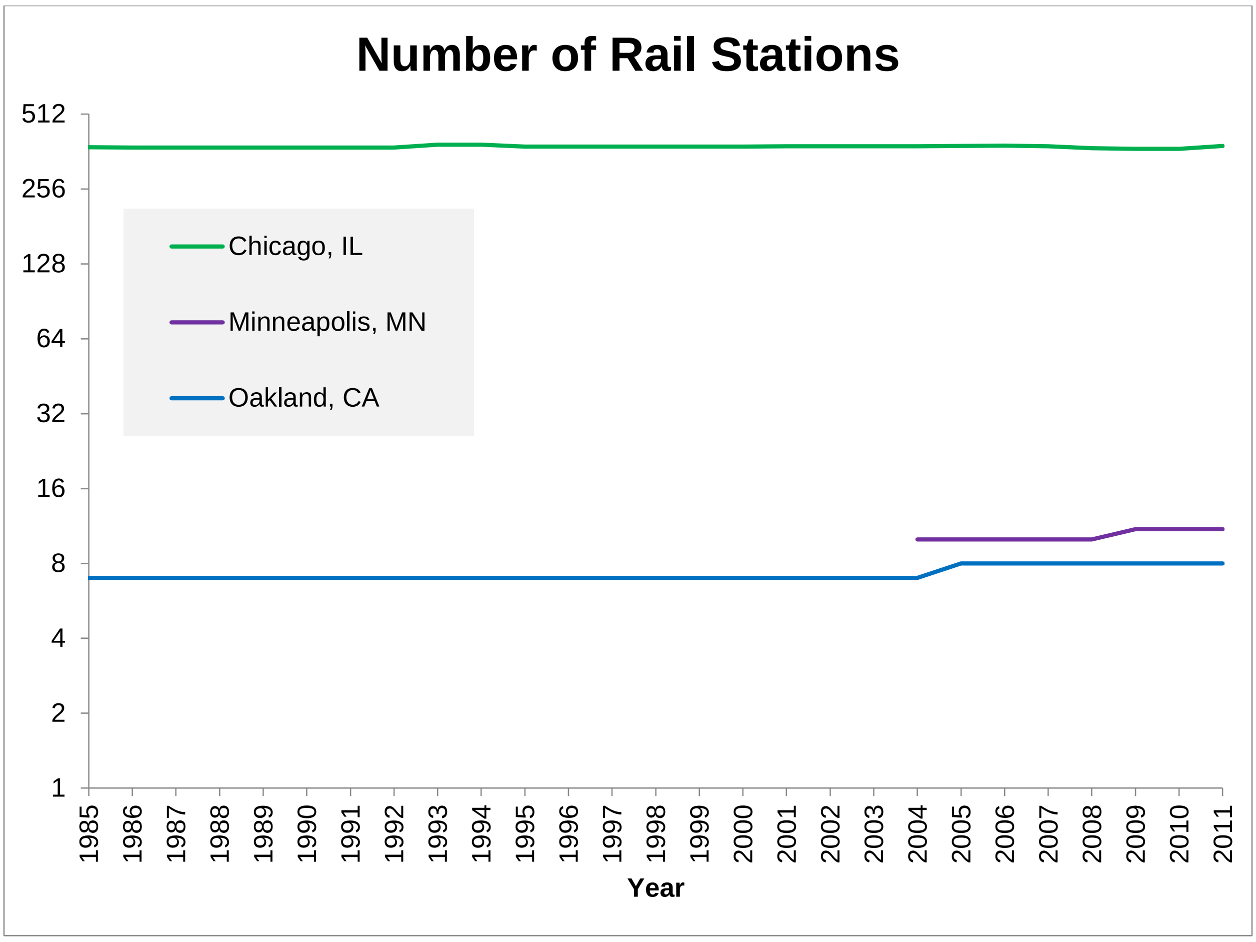

Figure S8: Change in rail line length (km) between 1985 and 2011 by city in the CARDIA Four Cities field audit. No rail in Birmingham, AL. Green represents Chicago, IL; blue represents Oakland, CA; purple represents Minneapolis, MN; red represents Birmingham, AL.

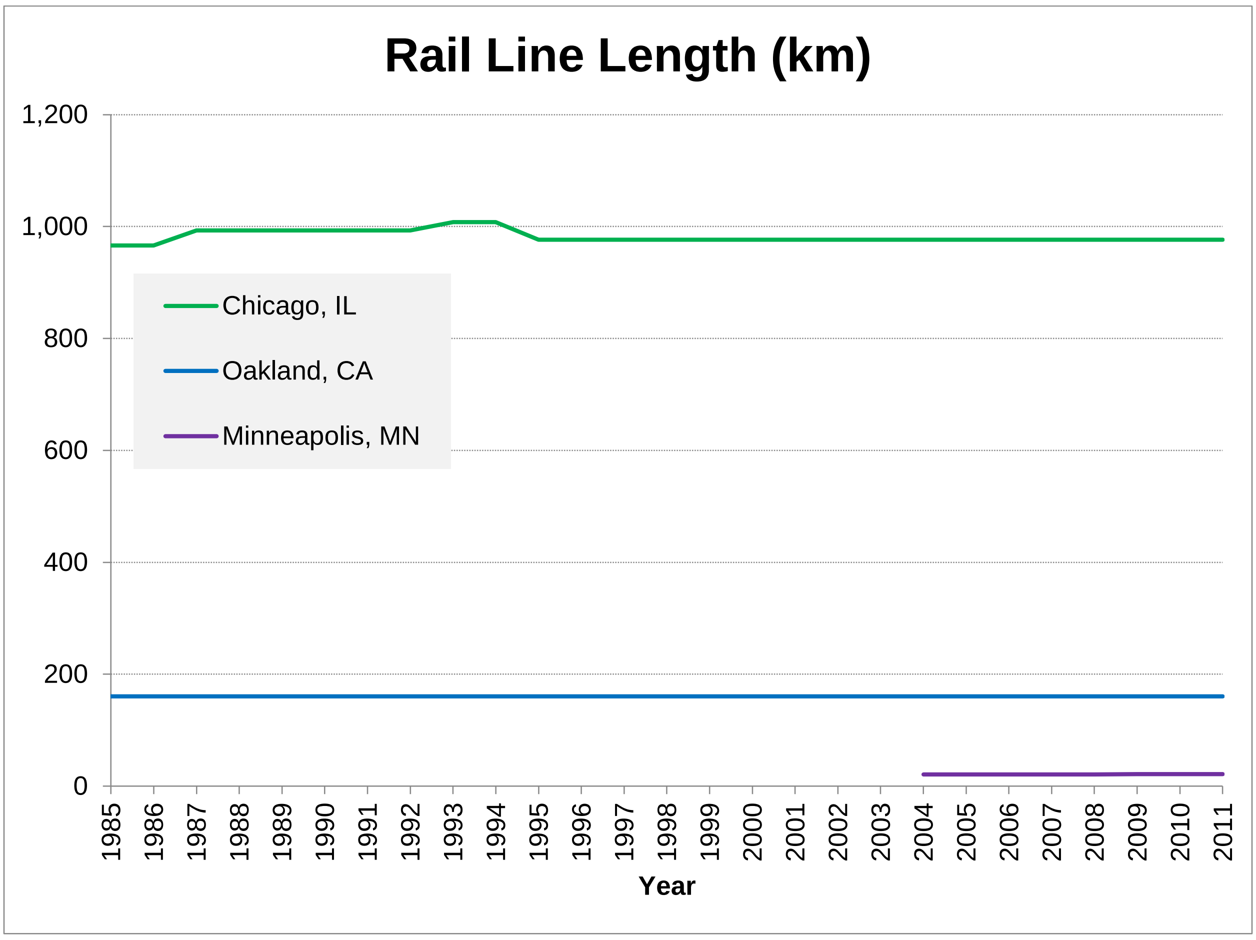

Figure S9: Change in park area (km^2) between 1985 and 2011 by city in the CARDIA Four Cities field audit. Green represents Chicago, IL; blue represents Oakland, CA; purple represents Minneapolis, MN; red represents Birmingham, AL.

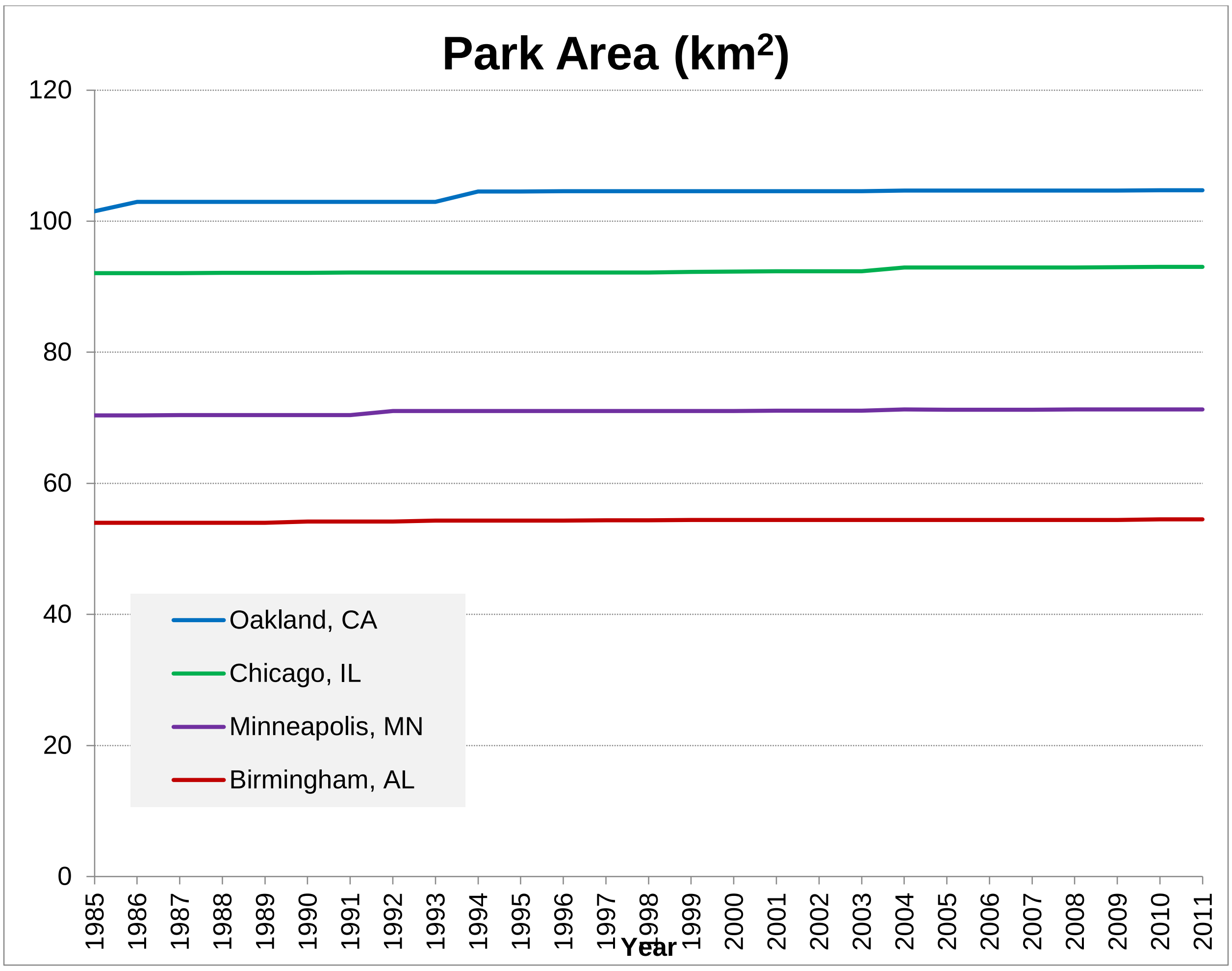

Supplement: Supplementary file 1 [file Data_Sheet_1.pdf]
